# High Intensity Resistance Exercise Training to Improve Body Composition and Strength in Older Men With Osteosarcopenia. Results of the Randomized Controlled Franconian Osteopenia and Sarcopenia Trial (FrOST)

**DOI:** 10.3389/fspor.2020.00004

**Published:** 2020-01-28

**Authors:** Wolfgang Kemmler, Markus Weineck, Matthias Kohl, Simon von Stengel, Jürgen Giessing, Michael Fröhlich, Daniel Schoene

**Affiliations:** ^1^Institute of Medical Physics, Friedrich-Alexander University of Erlangen-Nürnberg, Erlangen, Germany; ^2^Faculty of Medical and Life Sciences, Furtwangen University, Villingen-Schwenningen, Germany; ^3^Institute of Sports Science, University of Koblenz-Landau, Landau, Germany; ^4^Department of Sports Science, University of Kaiserslautern, Kaiserslautern, Germany

**Keywords:** high intensity, resistance exercise, sarcopenia, osteopenia, older men, fat free mass, muscle strength

## Abstract

Considerably decreased muscle mass and function are subsumed under “sarcopenia,” a geriatric syndrome. Dedicated exercise programs maintain muscle mass and function; however, due to the limited enthusiasm of older adults to exercise, it is important to generate low-threshold interventions for this vulnerable cohort. Thus, the primary aim of this study was to determine the effect of low volume/high intensity resistance exercise training (HIT-RT) combined with protein supplementation on body composition and strength in older men with sarcopenia and osteopenia (osteosarcopenia). Forty-three community-dwelling (cdw) older men (78 ± 4 years) with osteosarcopenia were randomly allocated to a consistently supervised HIT-RT (*n* = 21) or an inactive control group (CG, *n* = 22). HIT-RT scheduled a single set protocol with high intensity and effort applied twice a week for 36 weeks so far. Both groups were supplemented with Vit-D (800 IE/d), calcium (1,000 mg/d) and whey-protein (CG: 1.2 vs. HIT-RT: 1.5–1.7 g/kg/d). Study endpoints were body composition (dual-energy x-ray absorptiometry) and maximum isokinetic hip/leg-extensor strength (MIES) by leg-press. After 36 weeks, one participant who developed prostate cancer after inclusion in the study (HIT-RT) and two participants who lost interest (CG, HIT-RT) quit the study. Attendance rate for HIT-RT averaged 93 ± 5%. Total and thigh lean body mass (LBM) significantly (*p* < 0.001) increased in the HIT-RT and was maintained in the CG (*p* = 0.46 and 0.37). Differences between the groups for changes of total and thigh LBM were pronounced (*p* < 0.001; SMD d′ = 1.17 and 1.20). Total and abdominal body fat percentage decreased significantly in the HIT-RT (*p* < 0.001) and increased in the CG (*p* = 0.039 and *p* = 0.097). Intergroup differences were significant (*p* < 0.001; SMD: d′ = 1.35 and 1.28). Finally, MIES was maintained in the CG (*p* = 0.860), and improved significantly (*p* < 0.001) in the HIT-RT. Differences between the groups were significant (*p* < 0.001, SMD: d′ = 2.41). No adverse effects of the intervention were observed. In summary, the HIT-RT/protein protocol significantly affected body composition and strength in cdw men 72 years+ with osteosarcopenia. In the absence of negative side effects, the intervention was feasible, attractive and time effective. Thus, we conclude that supervised HIT-RT might be an exercise option for older men.

## Introduction

The loss of muscle mass and strength is an inevitable process during human adults' aging. In excess, this degradation is summarized under the term “sarcopenia” (Rosenberg, 1989), a geriatric syndrome (Cruz-Jentoft et al., 2010b) recently included in the ICD-10 GM^1^ code as a musculoskeletal disease (M62.84).

Resistance exercise combined with protein supplementation might be the most promising candidate to counteract age-related muscle loss (Hurley et al., 2011) and sarcopenia (Rosenberg, 1989). In summary, a recent umbrella review (Beckwee et al., 2019) found a “high quality of evidence” for a significant effect of exercise on muscle mass and strength in the area of sarcopenia prevention and treatment. However, considering the enormous heterogeneity of the underlying studies with respect to type, methods and composition of resistance exercise, it is difficult to derive precise exercise recommendations (Gentil et al., 2017). Apart from exercise-specific aspects, the low motivation and associated compliance with exercise of older adults (Rütten et al., 2009; Carlson et al., 2010) is problematic and needs to be taken into account when designing feasible exercise protocols.

Reviewing the literature, there is some evidence for higher effects of high intensity RT (HIT-RT) on muscle strength (Peterson et al., 2010; Steib et al., 2010; Csapo and Alegre, 2016) and a superior effect of higher volume RT (HV-RT) on muscle mass parameters (Peterson et al., 2011). Unfortunately, it is unlikely that older people with or at risk of sarcopenia will embrace the notion that they should exercise with high intensity and high volume to address muscle mass and strength. However, in a recent 16-week randomized controlled trial with sedentary middle-aged healthy men (Wittke et al., 2017), we observed similar (significant) effects on muscle mass and strength parameters when comparing a HV-RT vs. a time-efficient HIT-RT^2^; but only when HIT-RT was combined with protein supplements. However, we are not aware of “true” HIT-RT studies in community dwelling (cdw) older people, let alone in the vulnerable cohort of men with osteosarcopenia. Thus, the primary aim of the present study was to determine the effect of a time-efficient HIT-RT exercise protocol on body composition and strength in older people with osteosarcopenia. Further, safety, feasibility and attractiveness of our approach was also an important study feature.

Our primary hypothesis was that lean body mass would increase significantly in the HIT-RT group compared to the non-training control group. Our core secondary hypothesis was that (a) thigh lean mass and (b) hip/leg extensor strength as assessed on an isokinetic leg-press would increase significantly compared to the non-training control group. Another secondary hypothesis was that (c) total body fat and (d) abdominal fat would decrease significantly compared to the non-training control group. An experimental hypothesis was that HIT-RT would be safe, feasible and attractive for this cohort of older, community-dwelling (cdw) men with osteosarcopenia.

## Materials and Methods

### Study Design

FrOST (**Fr**anconian **O**steopenia and **S**arcopenia **T**rial) is an ongoing randomized controlled 18-month exercise project with a balanced parallel group design and two study arms. The study included community-dwelling men 72 years and older with sarcopenia and osteopenia. The key aims of the trial are (a) to evaluate the effect of high intensity resistance exercise training (HIT-RT) on bone mineral density and (b) to determine the effect of HIT-RT on sarcopenia parameters. The project was planned and initiated by the Institute of Medical Physics (IMP), University of Erlangen-Nürnberg (FAU), Germany. The University Ethics Committee of the FAU (number 67_15b and 4464b) and the Federal Bureau of Radiation Protection (BfS, number Z 5-2246212-2017-002) approved the trial. The study fully complies with the Helsinki Declaration “Ethical Principles for Medical Research Involving Human Subjects” (World_Medical_Association., 2013). All participants were able to participate in the project free of charge. After receiving detailed information, all study participants gave their written informed consent. The project was registered under ClinicalTrials.gov: NCT03453463. The current publication focuses on training induced changes of body composition and muscular strength during the first 36 weeks of the intervention (June 2018 to February 2019).

### Participants

**Figure 1:**
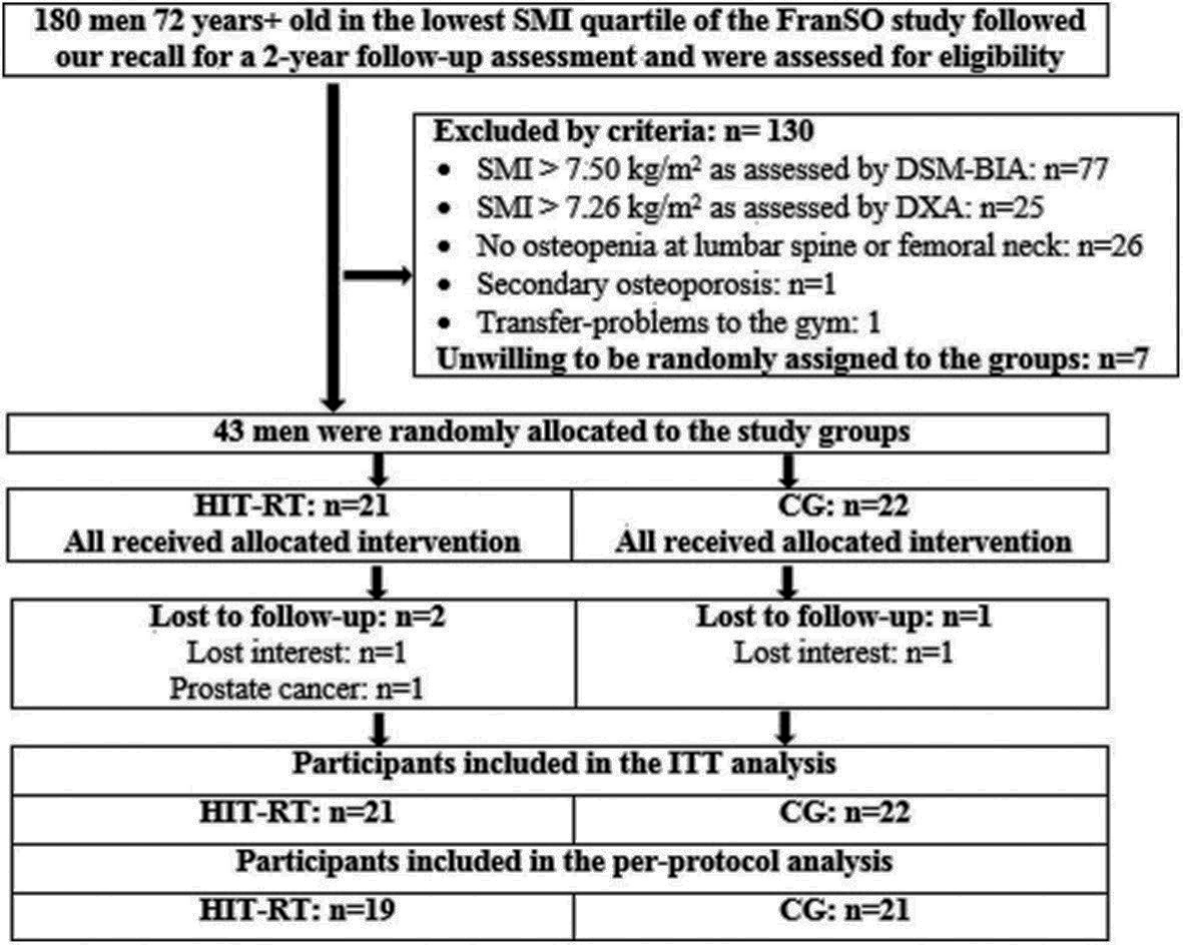
Participant flow through the study.

Participant recruitment was based on the Franconian Sarcopenic Obesity (FranSO) project (Kemmler et al., 2017a), a study conducted in 2016 to determine the prevalence of sarcopenia and sarcopenic obesity in Bavaria, Germany. Out of the entire cohort of 965 community dwelling men 70 years and older, participants from the lowest quartile for skeletal muscle mass index (SMI ≤ 7.78 kg/m^2^; *n* = 242) were invited to a two-year follow-up (FU) assessment in 2018. One hundred and eighty (180) men followed our invitation (Kemmler et al., 2019) and were further assessed for eligibility for the present study (Figure 1). After applying our exclusion criteria (a) amputations of limbs or cardiac pacemaker, (b) implementation of glucocorticoid therapy >7.5 mg/d during the last 2 years, (c) cognitive impairments with impact on the present assessment (Kemmler et al., 2019), (d) alcohol consumption of more than 60 g ethanol per day, (e) SMI >7.50 kg/m^2^ as determined by direct-segmental, multi-frequency Bio-Impedance-Analysis (DSM-BIA), the 103 men remaining were assessed for body composition and Bone Mineral Density (BMD) using Dual Energy x-Ray absorptiometry (DXA). Subjects were included in FroST when (a) Morphometric sarcopenia [SMI T-Score ≤ −2 SD (7.26 kg/m^2^), (Baumgartner et al., 1998; Cruz-Jentoft et al., 2010a)] and (b) osteopenia [BMD T-Score ≤ −1 SD, (WHO, 1994)] at the Lumbar Spine or the Proximal Femur was diagnosed. Exclusion criteria were specified as (a) secondary osteoporosis, (b) history of hip fracture and (c) resistance type exercise > 45 min/week. Applying the eligibility criteria left 43 men, who were willing to be randomly allocated either to an exercise (HIT-RT: *n* = 21) or control group (CG, *n* = 22). Figure 1 shows the participants' flow through the study.

### Randomization Procedures

Using three strata for SMI, there was a random and balanced assignment to the two study groups, consistently supervised HIT-RT (*n* = 21) or inactive control (CG: *n* = 22). In detail, the participants allocated themselves to the study group by drawing lots from a bowl. The lots themselves were placed in small opaque plastic containers (“kinder egg,” Ferrero, Italy). Lots were prepared by a person not involved in the present project, thus neither participants nor researchers knew the allocation beforehand. After the randomization procedure, the primary investigator (WK) enrolled participants and instructed them in detail about their study status and corresponding dos and don'ts.

### Blinding

We conducted a single blinded approach that focused on outcome assessors and test assistants only. Outcome assessors were unaware of the participants' group status (HIT-RT or CG) and were not allowed to ask, either. Furthermore, participants were blinded for protein supplementation (protein dose).

### Study Procedure, Intervention

All participants were asked not to change their physical activity and exercise habits outside the present study intervention. Further, apart from supplementation prescribed by the study protocol, all the men were requested to maintain their dietary habits.

Compliance with the exercise protocol was checked by instructors who consistently supervised the exercise sessions, by the gym's chip card system and by analyzing the training records completed by the participants of the HIT-RT. Compliance with the protein, Vit-D, and calcium supplementation was monitored by (a) the corresponding distribution records, (b) biweekly phone calls by the research assistant responsible for the nutritional analysis (c) personal interviews conducted by the primary researcher (WK) at 36-week follow-up. The intervention started in June 2018.

### Intervention: Resistance Exercise

The exercise program was performed in a well-equipped, centrally located gym (Kieser Training, Erlangen, Germany) that could be easily reached via public transport. Three training sessions/week (Monday, Wednesday, Friday), each between 8:30 and 10:00 a.m., were offered, all of which were consistently supervised and led by licensed instructors. However, the exercise protocol consistently only scheduled two sessions/week, thus, participants were free to attend their preferred training dates. All the participants of the HIT-RT were provided with detailed training records that prescribed exercises, number of sets, number of repetitions (reps), movement velocity and relative exercise intensity defined as “non-repetition maximum” (nRM), “(self-determined) repetition maximum” (RM) or “complete momentary muscular failure” (Steele et al., 2017). Absolute intensity of the exercise was regulated by prescribing a range of reps (i.e., 8–10 reps) and the corresponding degree of (incomplete) work to failure^3^ (Kemmler et al., 2015, 2016). The exercise protocol for the first 36 weeks was structured into four periods of eight (phase 2, 3, 4) and 12 (phase 1) weeks with different aims. Before each new training period, joint meetings were conducted to inform participants (HIT-RT) about the aims and contents of the following phase, to request feedback about the previous phase and to discuss issues related to exercise and supplementation.

#### Phase 1

The first 4 weeks of the project were dedicated to briefing, familiarization and learning the proper lifting technique. The next 8 weeks of conditioning with high emphasis placed on the adequate proportion of varying repetitions and corresponding loads considering the prescribed work to repetition maximum (Steele et al., 2017) constituted the second part of this phase. Fourteen exercises (latissimus front pulleys, rowing, back extension, inverse fly, bench press, shoulder press, lateral raises, butterfly with extended arms, crunches, leg press, leg extension, leg curls, leg adduction, and abduction) conducted on resistance devices (MedX, Ocala, FL, USA) were applied in phase 1. In each of the two sessions/week, 12 out of the 14 exercises were prescribed. Participants were free to select the order of the exercises. Using nearly the full range of motion, one to two sets of 8–15 reps, with a time under tension/rep of 2 s concentric, 1 s isometric and 2 s eccentric (2 s-1 s-2 s) to incomplete work to failure were scheduled in phase 1. Breaks/rest pauses were specified as 90 s (between sets) and 120 s (between exercises).

#### Phase 2

We introduced the single set approach (i.e., one set per exercise) structured in two linearly periodized 4-week phases, with each fourth week as a recovery week with low effort. The number of reps linearly decreased from 15 to 18 reps/set/session in the first and 5th week to 7–10 reps/set/session in the 3th and 7th week. We carefully introduced four new exercises (calf raises, hip extension, pull-overs, lateral crunches), and prescribed 14–15 exercises/session from the pool of 18 recognized exercises. Using a “repetitions in reserve” (RIR) approach (Zourdos et al., 2016), we prescribed incomplete work to failure. In other words, participants were requested to choose a load that ensured a repetition maximum −1 rep or −2 reps. Consistently, breaks of 90 s intermitted the exercises. Strong emphasis was placed on variation of the movement velocity ranging between 4 s-1 s-4 s per rep/session and 1 s-1 s-2 s per rep/session, still without explosive movement during the concentric phase.

#### Phase 3

We continued the training protocol reported for phase 2, using a slightly lower range of repetitions (6–8 to 12–15 reps; see above), and introduced explosive movement velocity during the concentric phase of the dynamic movement. From phase three on, about 30% of the sets/sessions were conducted with explosive movement. However, exercises with increased injury risk (e.g., back extension) were consistently conducted with moderate to low velocity. Further, we introduced the repetition maximum (RM) approach (Steele et al., 2017)^4^ in phase 3. Starting with four (core) exercises to be executed to repetition maximum during week 1, we increased the number of exercises to be conducted to RM to eight (core) exercises in week seven. Breaks between the exercises were 90 s (nRM) to 120 s (RM).

#### Phase 4

We also introduced the superset approach. One training session focused on supersets for the same or related muscle groups (e.g., upper back or knee extensors), the second session/week focused on opposed muscle groups i.e., agonist and antagonist (e.g., knee extensors on leg press and knee extensor on leg curls). A maximum of three exercises were included in a superset sequence. The number of supersets was increased from 2 to 4 during the first 3 weeks of phase 4. Rest pauses between exercises of a superset were in the range of 30–45 s. Breaks between the supersets or exercises not included in a superset row were 2 min.

### Protein Supplementation

Protein supplementation was based on 4-day diet records conducted at baseline and after 6 month (see below). We intended a total protein intake of 1.5–1.7 g/kg body mass/d in the HIT-RT and a corresponding intake of 1.2 g/kg body mass/d (Bauer et al., 2013) in the CG. Participants with a dietary protein intake <1.5 g/kg/d (HIT-RT) or <1.2 g/kg/d (CG) were provided with protein supplements. The protein powder used in FrOST (Active PRO80, inkospor, Roth, Germany) consists of whey protein with a chemical score of 156. One hundred grams contain 80 g of protein (10.4 g of Leucin), 5 g of carbohydrates and 1.8 g of fat resulting in a calorific value of 362 kcal/100 g protein powder. Furthermore, a 25 g/portion of the protein powder included 300 mg of calcium. Participants were requested to take the prescribed dose accurately on a daily base and to split doses higher than 30 g/d. We suggested mixing the protein power with low fat milk when applicable (or possible) in order to increase the participants' dietary calcium intake.

### Vit-D/Calcium Supplementation

Vitamin-D supplementation was based on 25 OH Vitamin-D 3 (25-OH D3) levels as assessed by blood samples at baseline (see below). Men ≤75 nmol/l (*n* = 37) were asked to supplement two units of 2,500 IE each, twice a week (i.e., 10,000 IE/week); participants ≤100 nmol/l (*n* = 4) were requested to take 5,000 IE/week (2 × 2,500 IE/d, once a week).

We intended to realize a calcium intake of about 1,000 mg/d in all participants (DVO, 2017). We calculated the amount of dairy dietary calcium intake based on dietary calcium questionnaires provided by the Rheumaliga Suisse. After considering the calcium provided by the protein powder, we prescribed the additionally required daily calcium to be taken as calcium capsules (Sankt Bernhard, Bad Dietzenbach, Germany). Each capsule contained 625 mg of calcium-carbonate with 250 mg of pure calcium.

### Study Outcomes

#### Primary Outcome

- Changes of Lean Body Mass (LBM) from baseline to 36-week follow-up as determined by Dual Energy X-ray Absorptiometry (DXA).

#### Secondary Outcome

- Changes of thigh (i.e., upper leg) LBM from baseline to 36-week follow-up as determined by DXA.
- Changes of maximum isokinetic hip/leg extensor strength (MIES) from baseline to 36-week follow-up as determined by an isokinetic leg press.
- Changes of total body fat [%] from baseline to 36-week follow-up as determined by DXA.
- Changes of abdominal body fat [%] from baseline to 36-week follow-up as determined by DXA.

#### Experimental Outcome

- Drop-out rate, attendance, adverse effects.

### Changes of Trial Outcomes After Trial Commencement

No changes of trial outcomes were made after trial commencement. However, due to a technical defect at 28 week FU, body composition assessment by DXA was determined after 36 weeks instead of the initially intended 28 weeks. Thus, the 36-week assessment can be considered as an originally unscheduled follow-up.

### Assessments

All assessments were conducted during a rest week. Further, participants were asked to restrain from intense physical activity and exercise 48 h pre-assessment. Overall, great emphasis was placed on the standardization of the tests including consistent verbal test descriptions. Baseline (BL) and FU assessments were performed using the identical consistently calibrated devices, in exactly the same setting and at the same time of the day (±90 min).

### Outcome Parameters as Assessed at Baseline and After 36 Weeks

Height was measured using a stadiometer (Holtain, Crymych Dyfed., Great Britain) and body mass was determined via direct-segmental, multi-frequency Bio-Impedance-Analysis (DSM-BIA; InBody 770, Seoul, Korea). Body composition was determine using both DSM-BIA and Dual Energy X-Ray Absorptiometry (DEXA, QDR 4500a, Discovery-upgrade, Hologic Inc., Bedford, USA), however in this publication we report the DXA results only. In order to standardize the BIA (and DXA) assessment, we consistently used the same test protocol, which includes minor physical activity 8 h pretest and 15 min of rest in a supine position immediately before the BIA assessment. Further, all participants were provided with written specifications about dos and don'ts. Apart from restraining from intense physical activity, participants were provided with standardized nutritional recommendations for the two meals prior to the BIA and DXA assessments^5^. However, due to the exercise tests that were applied at the same time and the aspect that not all participants were assessed in the morning, we restrained from an overnight fast.

Based on a total body DXA scan (i.e., excluding the skull) applying the manufacturer's standard total body segmentation protocol, subtotal Lean Body Mass (LBM) was measured using the “compare mode” at follow-up, which exactly reproduced the area and placement of the baseline assessment. Appendicular skeletal muscle mass (ASMM) was computed using the results of the upper and lower limbs provided by the standard specification of the DXA device. Skeletal Muscle Mass Index (SMI) was calculated as ASMM/body-height (kg/m^2^) (Baumgartner et al., 1998). The region of interest for LBM of the thigh (or upper legs) was segmented between the lower edge of the ischium and the lower edge of the femur. Relative total Body Fat (%) refers to the fat mass of the total body adjusted for body mass. The region of interest for abdominal body fat was segmented between the lower edge of the twelfth rib and the upper edge of the iliac crest. All baseline and follow-up scans were conducted and analyzed by the same research assistant.

Maximum isokinetic strength of the hip and leg extensors was tested using an isokinetic leg press (CON-TREX LP, Physiomed, Laipersdorf, Germany) at BL, after 28 and after 36 weeks. Maximum bilateral hip/leg extension was performed in a sitting, slightly supine position (15°), supported by hip and chest straps. Range of motion was selected between 30° and 90° of the knee angle, with the ankle flexed 90° and positioned on a flexible sliding footplate. The standard default setting of 0.5 m/s was used. After familiarization with the movement pattern, participants were asked to conduct five repetitions with maximum voluntary effort (“push as strongly as possible”). Participants conducted two trials intermitted by 2 min of rest. We included the highest value for hip/leg extension in the data analysis.

During the BL visit, participants completed a standardized questionnaire (Kemmler et al., 2004a) that asked for various aspects including (a) demographic parameters, (b) diseases, (c) medication, (d) operations, (e) physical limitations, (f) falls, injuries and low trauma fractures within the last year and (f) lifestyle, including physical activity and exercise (Kemmler et al., 2004b). After 36 weeks of intervention, all the participants conducted a comparable questionnaire particularly in order to detect changes that may affect our study endpoints. Prior to the visits, participants were required to list their medications and diseases in order to ensure accuracy of the responses. In close cooperation between primary investigator and the corresponding participants, the completed questionnaires were carefully checked for consistency, completeness and accuracy.

### Baseline Characteristics

Although listed in Table 1, the following parameters were not addressed by the 36-week FU assessment, thus only a short description will be provided.

**Table 1:** Baseline characteristics of the participants of the CG and HIT-RT group.

**Variable**	**CG (*n* = 22)**	**HIT-RT (*n* = 21)**	***p***
Age [years]	79.2 ± 4.7	77.8 ± 3.6	0.262
Body Mass Index [kg/m^2^]	24.5 ± 1.9	25.0 ± 3.0	0.515
More than 2 diseases [*n*]^a^	12	10	0.826
Physical activity [Index]^b^	4.15 ± 1.53	4.45 ± 1.32	0.490
Regular exercise ≥1x week [*n*]	5	5	0.931
SMI [kg/m^2^]	6.89 ± 0.31	7.01 ± 0.27	0.671
Handgrip-strength [kg]	30.0 ± 4.3	30.7 ± 5.1	0.675
Habitual gait velocity [m/s]	1.26 ± 0.15	1.25 ± 0.17	0.703
ALEF, LLFDI [Index]^c^	1.73 ± 0.82	1.87 ± 1.05	0.646
25 (OH)D [nmol/l]	54.0 ± 21.1	43.8 ± 17.5	0.126
Energy Intake [kJ/d]^d^	9,393 ± 2419	8,836 ± 1706	0.407
Protein Intake [g/kg/d]^d^	1.29 ± 0.34	1.10 ± 0.25	0.043
Smokers [*n*]	4	3	0.959

a Using the ICD-10 based disease cluster of Schafer et al. (2010);

b scale from (1) very low to (7) very high (Kemmler et al., 2004b);

c “Advanced Lower Extremity Function,” Late Life Function Disability Instrument (McAuley et al., 2005): [scale from (1) “no problem” to (5) “impossible”];

d *as determined by a 4-day dietary record*.

Habitual gait speed was performed using the 10 m protocol (Peters et al., 2013). Tests were performed twice without any walking aids using regular shoes. After the standardized instruction “walk at a speed just as if you were walking along the street to go to the shops,” participants started in an upright position 3 m before the first and stopped 2 m after the second photo sensor (HL 2-31, TagHeuer, La Chaux-de-Fonds, Suisse).

Handgrip strength tests were performed while standing upright, arms down by the side (Mathiowetz et al., 1984) with 30 s rest between the 3 trials for each hand. Handgrip width was adjusted individually to participant hand size. Participants were asked to “squeeze as strongly as possible.” The trial with the highest result of the dominant hand was included in the present analysis.

In order to assess changes of self-rated physical performance, we used the abridged version of the Late Life Function and Disability Instrument (LLFDI; McAuley et al., 2005).

Blood was drawn after an overnight fast between 7:00 and 9:00 in the morning in a sitting position from an antecubital vein. Serum samples were centrifuged for 20 min at 3,000 RPM and analyzed by the “Zentrallabor” of the University Hospital, FAU Erlangen-Nürnberg. Serum concentrations of 25(OH)D were measured using a Roche Modular E170 Analyzer and an electro-chemiluminescence immunoassay (ECLIA; Roche Diagnostics, Penzberg, Germany).

### Diet Records

Four-day diet records were conducted by all the participants at BL and after 28 weeks^6^. Participants were carefully briefed and instructed on how to keep the records. Participants were provided with diet records [Freiburger Nutrition Record (nutri-science, Hausach, Germany)], structured in food categories that included more than 250 food products along with the corresponding weight units. The Freiburger Nutrition Record also included a tally-list of how often the food products were consumed. The completed diet records were consistently analyzed by the same research assistant.

Participants were asked to protocol 3 weekdays and one weekend day representative for their nutritional habits. Results of the diet records were carefully discussed with the participants. In cases of unrealistic results (e.g., energy intake <1,000 kcal/d or >3,500 kcal/d), another diet record based on more representative days had to be completed.

### Sample Size

Considering (changes of) the study endpoint Bone Mineral Density (BMD) as “more discrete” compared to muscle mass parameters, the sample size calculation for the FrOST project was based on BMD at the lumbar spine as determined by quantitative computed tomography (QCT). This parameter was not addressed by the 36-week assessment however, due to the expected slower exercise-induced changes of bone compared to muscle tissue. Thus, we opted to report the factual statistical power of our sample size here (HIT-RT: *n* = 21 vs. CG: *n* = 22) as calculated by the approach reported above. Assuming an effect (Δ-HIT-RT vs. Δ-CG) on LBM of 0.9 ± 1.0 kg (Kemmler et al., 2017b, 2018)^7^ and applying a *t*-test based sample size calculation, the sample size of 21 participants/group corresponds to a 83% power (1-β) at a type-I-error of alpha = 0.05.

### Statistical Analysis

We calculated an intention to treat (ITT) analysis that included all participants randomly assigned to the two study arms (HIT-RT vs. CG) regardless of their compliance or loss to follow-up. A per protocol analysis that included only participants with complete datasets was also conducted. Multiple imputation (ITT) was calculated using R statistics software (R Development Core Team Vienna, Austria) in combination with Amelia II (Honaker et al., 2011). The full data set was used for multiple imputations, with imputation being repeated 100 times. Imputation worked well in all cases as confirmed by over imputation diagnostic plots (“observed vs. imputed values”) provided by Amelia II. Normal distribution of the data listed in Tables 2–4 was checked by statistical (Shapiro–Wilks) and graphical (qq-plots) tests. Based on the corresponding results, all the primary and secondary outcomes addressed here were analyzed by dependent *t*-tests for within group changes. To identify group differences, pairwise *t*-test comparisons (HIT-RT vs. CG) with pooled SD were applied. Alternatively, a repeated measures ANOVA (group by time interaction) was calculated within the per protocol analysis. We consistently applied 2-tailed tests, significance was accepted at *p* < 0.05. We also calculated Standardized Mean Difference (SMD) according to Cohen (d′; Cohen, 1988) to analyze effect sizes.

**Table 2:** Baseline data and changes of LBM in the CG and HIT-RT and corresponding between group differences.

	**CG**	**HIT-RT**	**Difference**	***p*-value**
	**MV ± SD**	**MV ± SD**	**MV (95% CI)**	
**Lean body mass [kg]**				
Baseline	43.19 ± 4.84	44.93 ± 4.66	–	0.239
Changes	−0.19 ± 0.92	1.26 ± 1.50^***^	1.45 (0.65 to 2.26)	<0.001

*** *p < 001*.

## Results

Table 1 displays participant baseline characteristics. As expected, habitual exercise participation was low in both groups. Most BL characteristics were evenly distributed between the CG and the HIT-RT. However, body height (not listed in Table 1) and relative protein intake (Table 1) differ significantly between the groups. Although no participant used protein powder, baseline protein intake of 15 participants of the CG was equal or higher than our intended protein supplementation of 1.2 g/kg/d.

Altogether three participants were lost to 36-week FU (Figure 1). One man retrospectively stated that he had preferred the CG and withdrew immediately after being randomly assigned to the exercise group. Another participant of the exercise group was unable to attend follow-up assessments for medical reasons. One participant of the CG lost interest and withdrew from the study. No participant of the HIT-RT group quit the study for intervention-related reasons. Further, no resistance training induced complaints or unintended side effects were reported. Average training attendance as determined by the gym chip card system was 65.3 of a maximum of 70 sessions (attendance rate 93 ± 5%; range: 83–100%). The average time expended for an exercise session was 48 ± 8 min.

Based on our distribution logs, biweekly phone calls and personal interviews at 36-week follow-up assessments, compliance with the protein, Vit-D and calcium supplementation was satisfactory. However, three participants (HIT-RT: *n* = 2, CG: *n* = 1) reported problems (constipation, diarrhea, nausea) from taking their individually calculated protein doses of 25–40 g/d of whey protein. After changing to whey isolate, only one of these subjects was still unable to realize the prescribed dose. Blinding of protein supplementation was successful in that both groups felt they were getting the same amount of protein.

With respect to our experimental hypothesis, we thus conclude that our combined HIT-RT/protein supplement approach was safe, feasible and attractive for this male cdw cohort with osteosarcopenia and low affinity for exercise (Table 1).

Changes in the primary study endpoint are listed in Table 2. Based on comparable baseline data, LBM improved significantly (*p* < 0.001) in the HIT-RT and deteriorated slightly in the CG (*p* = 0.46). Differences between the groups for changes of LBM were significant (*p* < 0.001; SMD d′ = 1.17; Table 2). The additionally conducted Per-Protocol Analysis was very close to these data (*p* < 0.001; SMD: d′ = 1.19). Thus, we confirmed our primary hypothesis that lean body mass increases significantly following HIT-RT compared to the non-training control group.

In parallel, thigh lean mass improved significantly in the HIT-RT (*p* < 0.001) and decreased slightly (*p* = 0.37) in the CG. Differences between the groups were significant (*p* < 0.001; SMD: d′ = 1.20; Table 3).

**Table 3:** Baseline data and changes of thigh muscle mass and hip/leg extensor strength in the CG and HIT-RT and corresponding between group differences.

	**CG**	**HIT-RT**	**Difference**	***p*-value**
	**MV ± SD**	**MV ± SD**	**MV (95% CI)**	
**Thigh lean body mass [kg/g]**				
Baseline	6.88 ± 0.62 kg	7.17 ± 0.92 kg	–	0.243
Changes	−37 ± 233 g	242 ± 231 g^***^	259 (114–405)	<0.001
**Maximum hip/leg extension strength (Leg press) [*****N*****]**				
Baseline	1,746 ± 389	1,620 ± 497	–	0.368
Changes	5 ± 138	451 ± 223^***^	446 (328–564)	<0.001

*** *p < 0.001*.

Based on slightly lower baseline values, no relevant changes (*p* = 0.860) of hip/leg extension strength were observed in the CG, while leg press performance increased by 28.3 ± 16.6% (*p* < 0.001) in the HIT-RT. Differences between the groups were significant (*p* < 0.001, SMD: d′ = 2.41) in favor of the HIT-RT.

Total (TBF) and abdominal body fat (ABF) of the HIT-RT and CG were comparable at BL (Table 4). Both parameters decreased significantly in the HIT-RT (*p* < 0.001) and increased in the CG (TBF: *p* = 0.039 and ABF: *p* = 0.097). Between-group differences were significant for TBF and ABF (*p* < 0.001; SMD: d′ = 1.35 and 1.28; Table 4) in favor of the HIT-RT.

**Table 4:** Baseline data and changes of total and abdominal body fat in the CG and HIT-RT and corresponding between group differences.

	**CG**	**HIT-RT**	**Difference**	***p*-value**
	**MV ± SD**	**MV ± SD**	**MV (95% CI)**	
**Total body fat [%]**				
Baseline	33.57 ± 3.92	34.24 ± 6.20	–	0.681
Changes	0.89 ± 1.84^*^	−1.76 ± 2.08^***^	2.65 (1.39–3.91)	<0.001
**Abdominal fat [%]**				
Baseline	37.48 ± 5.94	37.21 ± 6.54	–	0.892
Changes	0.92 ± 2.98	−2.27 ± 1.89^***^	3.18 (1.56–4.82)	<0.001

* p < 005;

*** *p < 001*.

In summary, we thus confirmed our secondary hypothesis that (a) thigh lean mass, (b) leg extensor strength, (c) total body fat, (d) abdominal fat and isokinetic leg-press improve significantly following HIT-RT compared to the non-training control group.

Of importance, we did not observe any changes in potentially confounding variables that might have affected our results. Upon written and verbal request, participants did not report relevant changes (*p* ≥ 0.487) of habitual physical activity or exercise outside the study protocol. Apart from the participant with prostate cancer, no participant listed relevant changes in medications, diseases, musculoskeletal injuries or cardiometabolic events. Further, no period ≥2 weeks of diseases or disease-related inactivity were recorded. Based on the results of the 28-week dietary record, energy (CG: 32 ± 553 vs. HIT-RT: 53 ± 680 kJ/d, calcium (−37 ± 251 vs. −19 ± 199 mg/d) and protein intake (2.1 ± 12.9 vs. 3.5 ± 16.4 g/d) did not change (*p* ≥ 0.388) or differ between the groups (*p* ≥ 0.251).

## Discussion

This study is the first randomized controlled exercise trial to demonstrate the (time) effectiveness, safety and attractiveness of a consistently supervised HIT-RT protocol combined with moderately dosed protein supplementation in older cdw men with low muscle and bone mass. Unlike most other researchers in the field of sarcopenia, we focused mainly on changes in muscle mass parameters during the first 36 weeks of the study. We consider the role of muscle mass to be highly relevant in general, independently of its functional impact, but all the more so for older people. Apart from its prominent role on thermoregulatory control in cold and hot environmental conditions (Kenney and Buskirk, 1995; Blatteis, 2012; Balmain et al., 2018; Payne et al., 2018), muscle mass plays a vital role in energy intake control, thermogenesis and resting metabolic rate and thus may be of outstanding relevance for combating obesity (Clarke and Henry, 2010; Dulloo et al., 2017). Further, low muscle mass is associated with cardiometabolic risk factors ((Kim and Choi, 2015; Prado et al., 2018; Mesinovic et al., 2019), chronic inflammation (Minn and Suk, 2017; Suyoto and Aulia, 2019), metabolic syndrome (Zhang et al., 2018); diabetes (Srikanthan and Karlamangla, 2011; Son et al., 2017; Mesinovic et al., 2019), and white matter brain changes/silent infarction (Minn and Suk, 2017)^8^. Moreover, low muscle mass is associated with higher surgical and post-operative complications, and shorter survival (Prado et al., 2018). Another reason for our focus on muscle mass was that changes in muscle mass in older people were less prominent compared to strength changes (Stewart et al., 2014; Borde et al., 2015). In fact, exercise-induced effects of muscle mass/LBM in older adults were on average moderate at best (Stewart et al., 2014; Beckwee et al., 2019)^9^. Indeed, it is well documented that due to the blunted hypertrophic response (Phillips, 2009; Kirby et al., 2015) increases in muscle mass parameters are considerably lower compared to younger cohorts (Peterson et al., 2011). Thus, developing a protocol that relevantly increase muscle mass is much more challenging compared to triggering increases in muscle strength.

In summary, our 8-month periodized, consistently supervised and guided HIT-RT protocol combined with whey protein supplementation demonstrated a significant 3.3% net gain (1.45 kg; Table 2) in lean body mass. A comparable effect was observed for thigh lean mass (3.7%; Table 3). Reviewing the present literature, we rank this finding in the upper range of corresponding studies (review in Peterson et al., 2011; Stewart et al., 2014; Beckwee et al., 2019). One may argue that our results on LBM are higher due to the adjuvant protein supplementation. However, in their umbrella review on exercise and sarcopenia, Beckwee et al. (2019) did not recommend adjuvant nutritional supplementation. On the other hand, two meta-analyses (Cermak et al., 2012; Finger et al., 2015) and further more specified studies with older adults (Bauer et al., 2015; Rondanelli et al., 2016) reported significant (additional) effects of protein supplementation in older people undergoing resistance exercise. Based on these findings and the aforementioned result that protein supplementation augments the effect of a HIT-RT on LBM to a HV-RT level (Wittke et al., 2017), we applied a combined exercise/protein protocol in the FranSO study (Kemmler et al., 2017b, 2018). FranSO was a foregoing RCT with an almost identical cohort of men that focused on whole-body electromyostimulation (WM-EMS) as an exercise intervention. After 16 weeks of exercise and whey protein supplementation (total intake: 1.7 g/kg/d), we observed significant effects on muscle mass, muscle CSA of the thigh, total and abdominal body fat and maximum hip/leg extensor strength, albeit considerably lower compared to the present protocol.

Declines in maximum knee extension strength might be the most crucial physical parameter for mobility limitation, disability, morbidity and mortality in older people (Visser et al., 2005; Newman et al., 2006; Roshanravan et al., 2017). In the present study, hip/leg extensor strength as determined by the functionally meaningful isokinetic leg press test increased by 28 ± 15%. Notably, we still monitored a significant increase between the 28- and the 36-week assessment (+60 ± 71 N). Overall, our results fit well with the data of Peterson et al. (2010), who included 51 treatment groups in his meta-analysis on exercise and strength development in older people (50–92 years). Peterson et al. (2010) reported an average 29% increase in hip/leg extension maximum strength as determined by leg press (32 kg; 95% CI: 28–36 kg).

We also observed significant effects on total and abdominal body fat with a pronounced decrease in the HIT-RT and a slight increase in the CG. In absolute terms, the net effect (HIT-RT vs. KG) of exercise on total body fat mass was 2.1 kg (95% CI: 1.1–3.1 kg; Table 4). Although we anticipated the favorable changes in the HIT-RT (Kemmler et al., 2017b; Wittke et al., 2017), we are in fact unable to explain the pronounced increase in the CG.

Considering the attractiveness of the HIT-RT/protein protocol as determined by attendance rates, our results (93 ± 5%) are in the upper range of (mainly shorter) studies (88 ± 8%) with people 60 years and older (Kohler et al., 2012).

So far (36 weeks), we have not observed any negative side effect of the HIT-RT intervention. This might be related to the 12-week briefing, familiarization and conditioning period, the careful progression of strain intensity and effort, and the training on safe resistance training machines in a gym with excellent supervision. Another factor discussed later might be that we did not focus on “work to momentary failure” (Steele et al., 2017).

In summary, we conclude that RT protocols conducted with low volume but high intensity, velocity and effort on resistance machines are feasible, safe und time-efficient (2 session/week × 50 min/session) exercise methods for older men to improve body composition and strength and thus fight sarcopenia and sarcopenic obesity.

Some limitations and features may decrease the understanding and/or validity of our trial. First of all, due to the technical failure of the DXA scanner immediately before the scheduled 28-week assessment, we conducted a 36-week follow up in order to determine muscle mass indices more accurately. At first sight, it might be confusing that we applied the majority of tests twice within 8 weeks; however, this approach was necessary to determine reliable results. We validated the maximum isokinetic strength of the hip/leg extensors and applied questionnaires and personal interviews to detect changes of variables that may affect our outcomes, however, we decided to do not apply the entire assessment battery used at 28-week FU. Second, one may argue that our HIT-RT approach is not a pure-bred HIT protocol (Gießing, 2008). Indeed, we did not apply the work to momentary failure (WMF) approach (Steele et al., 2017) characteristic for HIT-RT protocols, i.e., participants were not required to exercise until they were unable to properly complete the concentric portion of their current repetition. We skipped this aspect and continued with advanced techniques (e.g., supersets; later in Phase 5: “drop sets”). The main reason for omitting WMF was grounded in the feedback of the participants that WMF was a “red line” of exercise intensity which they did not want to cross. Third, it was not our goal to determine the additional effect of protein on our low volume, high intensity/high RT-protocol. Rather, we were looking to determine the combined effect of two low-threshold approaches applicable for a group of older people with low enthusiasm for conventional exercise. Fourth, one may argue that when applying “sarcopenia” as a rationale for the present exercise study, one ought to report dedicated parameters like appendicular skeletal muscle mass (ASMM) or skeletal muscle mass index (SMI). We focus on these specific sarcopenia parameters at the 28-week follow-up (Lichtenberg et al., 2019), however we had to apply DSM-BIA for the assessment of SMI. In order to generate an adequate comparison of our results on body composition with the present literature we opted to focus on LBM, a parameter more frequently used compared to ASMM and SMI. Fifth, our definition of sarcopenia focuses on the morphometric aspect of this geriatric syndrome. Further, we applied the older version of the EWGSOP definition and SMI cut-off (Baumgartner et al., 1998; Cruz-Jentoft et al., 2010a), simply because the study start was before the publication of the new EWGSOP II definition (Cruz-Jentoft et al., 2019). Sixth, drawing lots might not be the most sophisticated randomization strategy, but in recent studies we observed that personally drawing lots and thus randomized self-assignment to a group boosted acceptance for an initially non-favored study group. This is a very important aspect for increasing adherence and compliance in essentially non-blindable exercise studies. Seventh, we did not ask participants to abstain from food for >8 h that is recommended as a pre-assessment diet to properly determine fat mass or LBM by DXA or BIA (Tinsley et al., 2017). Although we consistently apply our dietary approach this proceeding however can be considered as a potential confounder. Eighth, although we placed great emphasis on supervision, monitoring and briefing of our HIT-RT protocol, we were not always convinced that participants properly applied our training schedule in practice. Finally, it is debatable whether results evaluated in the recent sample of older men with morphometric sarcopenia and osteopenia can be simply generalized to healthy older adults. However, considering the specific nature of sarcopenia, which includes aspects of inflammation (Kalinkovich and Livshits, 2017), mitochondrial abnormalities (Calvani et al., 2013), oxidative stress (Sullivan-Gunn and Lewandowski, 2013) i.e., factors that may rather decrease muscle response to exercise (Bowen et al., 2015), HIT-RT effects in healthy older men should be at least comparably high.

## Data Availability Statement

The anonymized data used to support the findings of this study are available from the corresponding author upon request.

## Ethics Statement

The studies involving human participants were reviewed and approved by The University Ethics Committee of the FAU, Krankenhausstrasse 12, 91052 Erlangen, Germany. The patients/participants provided their written informed consent to participate in this study.

## Author Contributions

WK, MW, MK, SS, JG, MF, and DS designed the study, completed data analysis and/or interpretation, and drafted the manuscript. JG and MF contributed to study conception, design, and revised the manuscript. WK accepts responsibility for the integrity of the data sampling, analysis, and interpretation.

### Conflict of Interest

The authors declare that the research was conducted in the absence of any commercial or financial relationships that could be construed as a potential conflict of interest.

## Footnotes

^1^ICD, International Classification of Diseases and Related Health Problems; GM, German Modification.

^2^Defined as single set RT with high intensity and effort (Gießing, 2008; Steele et al., 2017).

^3^For e.g., maximum effort minus 1–3 reps; defined as non-Repetition Maximum (nRM, Steele et al., 2017): “Set end point when trainees complete a pre-determined number of repetitions despite the fact that further repetitions could be completed.”

^4^Set end point when trainees properly complete the final repetition possible whereby if the next repetition was attempted they would definitely achieve momentary failure (Steele et al., 2017). However, according to the definition, sets conducted with high velocity were terminated when explosive velocity was no more possible.

^5^With ≈60% carbohydrate, 20% fat and 20% protein content. We further recommended a fluid intake of 2.5-3 l during the last 24 h, with 0.5 l 2–3 h prior to the BIA/DXA assessments.

^6^Considering the work load and due to the temporal proximity we did not repeat the dietary record at 36-week FU.

^7^…reported by a recent trial (Kemmler et al., 2017b, 2018) that applied Whole Body Electromyostimulation (WB-EMS) and Protein supplementation in older men. However, a comparison between HIT-RT and WB-EMS reported higher LBM-changes for HIT-RT.

^8^…as an independent risk factor for stroke.

^9^… considering that the individual studies included in these meta-analyses did not consistently focus on older people (Kelemen et al., 1986; Bemben et al., 2000; Teixeira et al., 2003).
